# Sustainable Hydrochars from Winery Waste for the Efficient Removal of Organophosphorus Pesticides and Synthetic Dye

**DOI:** 10.3390/ijms27072984

**Published:** 2026-03-25

**Authors:** Jelena Petrović, Marija Koprivica, Marija Milenković, Marija Ercegović, Tamara Lazarević-Pašti, Tamara Terzić, Vedran Milanković, Marija Simić

**Affiliations:** 1 Institute for Technology of Nuclear and Other Mineral Raw Materials, 86 Franchet d’Esperey St., 11000 Belgrade, Serbia; m.koprivica@itnms.ac.rs (M.K.); m.ercegovic@itnms.ac.rs (M.E.); m.petrovic@itnms.ac.rs (M.S.); 2 Vinča Institute of Nuclear Science, University of Belgrade, 11000 Belgrade, Serbia; marija.kojic@vin.bg.ac.rs (M.M.); lazarevictlj@yahoo.com (T.L.-P.); tamara.tasic@vin.bg.ac.rs (T.T.); vedran.milankovic@vin.bg.ac.rs (V.M.)

**Keywords:** waste valorization, hydrothermal carbonization, dual-stage modification, organophosphorus pesticide removal, ultrasonic stability

## Abstract

The removal of water pollutants, specifically the organophosphorus pesticides chlorpyrifos (CHP) and azinphos-methyl (AZM), as well as the dye Rhodamine B (RB), was investigated through the valorization of grape pomace, an abundant agricultural byproduct. For the first time, hydrochars derived from grape pomace were utilized as adsorbents for these contaminants following KOH activation (HCK) and pyrolysis at 400 °C (PHC). The study aimed to evaluate the adsorption performance, determine the optimal conditions, and elucidate the adsorption mechanisms. Physicochemical characterization using SEM, FTIR, BET surface area analysis, stability, and pH_PZC_ measurements revealed distinct differences in surface morphology, functional groups, porosity, and surface charge. Under optimized conditions, maximum adsorption capacities reached 751.0, 3.98, and 1.39 mg g^−1^ for RB, CHP, and AZM, respectively, on HCK, and 616.0 (RB), 30.10 (CHP), and 9.15 mg g^−1^ (AZM) on PHC, indicating that the selected hydrochars efficiently removed the investigated pollutants from water. Kinetic modeling demonstrated pseudo-first-order adsorption for RB and CHP on HCK and pseudo-second-order adsorption for AZM on HCK and all pollutants on PHC. Thermodynamic analysis confirmed that adsorption processes were spontaneous and favorable, with enhancements dependent on temperature. These findings suggest that HCK is particularly effective for cationic dyes, while PHC exhibits greater affinity toward organophosphorus pesticides, offering complementary applications and providing new mechanistic insights into hydrochar-based pollutant removal.

## 1. Introduction

The release of contaminated industrial effluents and the extensive application of agrochemicals are major sources of environmental pollution. Key pollutants include synthetic dyes and organophosphorus pesticides such as Rhodamine B (RB), chlorpyrifos (CHP), and azinphos-methyl (AZM), which are frequently detected in natural water sources (typical concentrations range from <0.1 to 10 mg L^−1^ for RB and <1 µg L^−1^ for pesticides) and thus serve as suitable model pollutants for removal studies [1]. These substances are usually detected in soil, water, and air as a result of industrial effluent discharge, surface runoff, and improper waste disposal [2,3,4,5,6]. RB is widely utilized in the textile industry mainly because it is inexpensive but highly efficient as a color, while CHP and AZM are commonly used in agriculture due to their effectiveness in pest control and crop protection. The persistence and toxicity of these compounds pose a significant hazard and health risk, including neurotoxicity, endocrine disruption, and potential carcinogenicity [7]. Due to their complex chemical structures and detrimental effects, there is an urgent need to develop efficient and sustainable remediation strategies [4,5]. In addition to adsorption-based techniques, recent research has focused on the development of multifunctional and environmentally responsive materials with enhanced separation, photocatalytic, and self-cleaning capabilities for wastewater treatment and environmental protection [8]. These studies highlight the global trend toward integrating surface engineering and hybrid material design to achieve improved stability, hydrophobicity, and pollutant removal efficiency under complex environmental conditions. Owing to the high cost, limited removal efficiency, and risk of secondary pollution associated with conventional treatment methods, adsorption has emerged as a promising and cost-effective alternative for remediation [3,4,9]. A variety of adsorbents, including activated carbons, clays, and zeolites, have been explored for this purpose [1,3,10]. Winery waste, particularly grape pomace, has attracted growing attention as a renewable and low-cost biomass resource due to its large annual production and rich composition of lignocellulosic compounds and polyphenols. Various strategies for its valorization have been investigated, including the extraction of bioactive compounds, production of biofuels and biogas, composting and soil amendment, and the development of functional materials [11,12]. These diverse utilization pathways highlight the potential of winery residues to serve as valuable secondary resources rather than waste.

However, recent research has focused on the utilization of bio-based adsorbents such as hydrochars - carbonaceous materials produced via hydrothermal carbonization (HTC) of waste biomass - due to their sustainability, cost-effectiveness, and tunable properties [13,14,15]. Among different biomass resources, grape pomace represents a particularly promising precursor for hydrochar production, enabling the simultaneous valorization of winery waste and development of efficient adsorbent materials for environmental remediation. HTC is a thermochemical process conducted under subcritical water conditions (180–280 °C) and autogenous pressure, that converts wet biomass directly into hydrochar without feedstock drying [5,16]. This method enables the valorization of diverse waste biomass sources, including agricultural residues and food waste, providing an energy-efficient and environmentally sustainable route for both waste management and the synthesis of novel adsorbents [16]. Via HTC, biomass is hydrolyzed, dehydrated, and polymerized, yielding a porous, carbonaceous material with heterogeneous surface functional groups that not only facilitate efficient adsorption of pollutants but also enable surface modifications to optimize adsorption performance [17]. Furthermore, the surface chemistry of hydrochar can be tailored via physical or chemical treatments to improve selectivity toward specific pollutants and optimize adsorption performance [17].

So far, several studies have demonstrated the potential of modified hydrochars for dye and pesticide removal. For instance, Hou et al. [18] utilized nitrogen-doped hierarchical carbons derived from glucose hydrochar for RB adsorption, achieving a maximum capacity of 5181 mg g^−1^. Similarly, Li et al. [19] showed that citric acid-treated iron-enriched sludge hydrochar exhibited excellent RB removal, while a Zea mays-based magnetic hydrochar composite adsorbed up to 97.4% of CHP [9]. Additionally, other chemical treatments such as H_2_O_2_, H_2_SO_4_, and NaOH have been successfully applied for the modification of biomass-derived adsorbents for pesticide removal [20,21,22]. However, most existing studies focus on well-established adsorbents, and comparative evaluations of different modification strategies on hydrochar derived from the same biomass feedstock are limited. Notably, no studies have reported the use of hydrochar for AZM adsorption, nor the application of grape pomace-derived hydrochars for the removal of RB, CHP, and AZM. Although previous reports achieved high adsorption capacities for RB and CHP using nitrogen-doped or magnetic hydrochars [18], comparable data for AZM are lacking, and grape pomace hydrochars have not been evaluated for these contaminants. To address this gap, this study systematically investigates the effect of KOH activation and pyrolysis on hydrochar structure, surface chemistry, and adsorption behavior, providing new mechanistic insights into pollutant removal. By synthesizing and modifying grape pomace-derived hydrochars via environmentally benign routes and evaluating their physicochemical properties, adsorption performance, and structural stability, this work demonstrates for the first time the effective removal of AZM using hydrochars and highlights the influence of different modification strategies on adsorption efficiency.

Hydrochars were produced from grape pomace (GP) through HTC at 220 °C, followed by modification via (1) alkaline activation with KOH and (2) post-HTC pyrolysis at 400 °C to yield pyro-hydrochar. These simple but effective modifications were selected to enhance the structural and surface properties of the hydrochars. In particular, chemical activation with KOH is known for its high efficacy and affordability, while pyrolysis is a chemically neutral process that does not use additional reagents and reduces the environmental impact. The hydrochars were examined via Scanning Electron Microscopy (SEM), Fourier-Transform Infrared spectroscopy (FTIR), Brunauer–Emmett–Teller (BET) surface area analysis, point of zero charge, and particle size distribution (PSD), and their adsorption behavior was systematically evaluated through kinetic, isotherm, and thermodynamic studies. The aim was to explore the impact of surface modification methods on their physicochemical and adsorptive characteristics and to identify the controlling mechanisms of adsorption. The study also investigates, for the first time, the ultrasonic stability of grape pomace-derived hydrochars, providing insight into their structural integrity under mechanical stress in aqueous media, as well as cost analysis. Overall, the findings highlight their potential as low-cost, sustainable adsorbents for water purification, supporting circular economy principles and environmental protection.

## 2. Results and Discussion

### 2.1. Characterization of Materials

**Figure 1:**
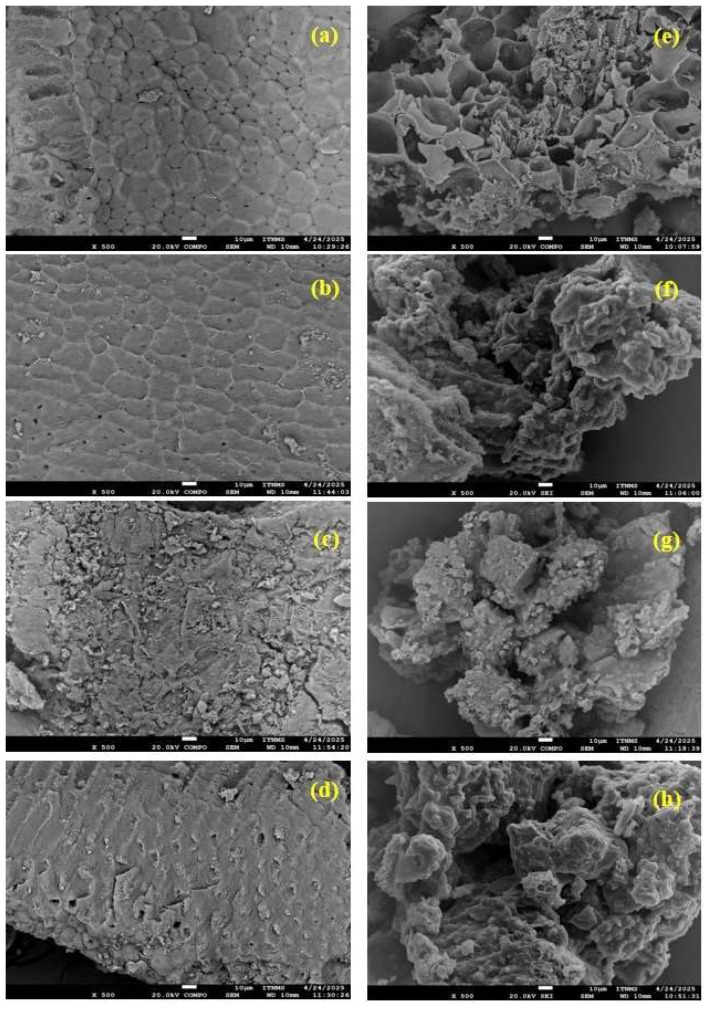
SEM images of HCK before (**a**) and after adsorption of CHP (**b**), AZM (**c**), and RB (**d**), and PHC before (**e**) and after adsorption of CHP (**f**), AZM (**g**), and RB (**h**); all images at 500× magnification, 20.0 kV, 10 µm scale bar.

**Figure 2:**
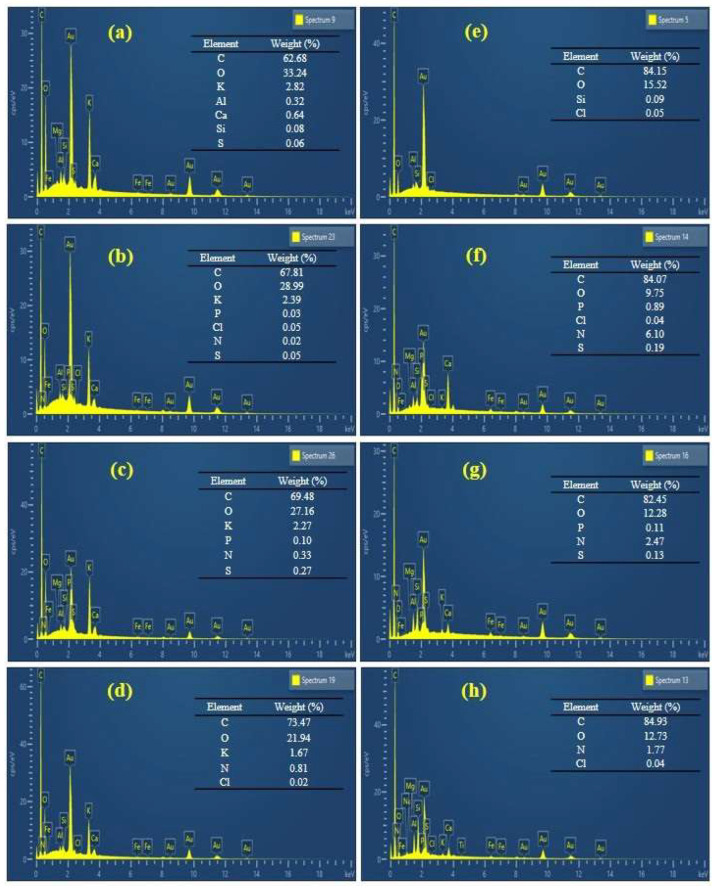
EDX spectra of HCK before (**a**) and after adsorption of CHP (**b**), AZM (**c**), and RB (**d**) and PHC before (**e**) and after adsorption of CHP (**f**), AZM (**g**), and RB (**h**).

Figure 1a–h represents SEM micrographs, while Figure 2a–h reveals EDX graphs of HCK and PHC hydrochars prior to and upon adsorption of selected pollutants. As illustrated in Figure 1e, PHC exhibits a distinctly layered, rough, and non-uniform surface, characterized by pronounced cracks, cavities, and fragmented regions. These features result from post-hydrothermal pyrolysis at 400 °C, which promotes devolatilization, carbon matrix degradation, and facilitates the breakdown of residual biopolymers [23]. In contrast, HCK (Figure 1a) exhibits a smoother and more uniform morphology with visible channels, attributed to milder KOH activation that induces surface cleaning without significant pore development [9,15]. After pesticide adsorption, a noticeable decrease in surface roughness, channels, and cavities is observed, especially for PHC. This indicates that pollutant molecules effectively bind to the material surfaces [5]. Similar behavior was also seen during the removal of RB with both adsorbents.

To verify the binding of pollutants to the two tested materials, EDX spectra (Figure 2a–h) were obtained and analyzed both before and after adsorption. In the EDX spectra of HCK and PHC prior to adsorption, the most dominant peaks correspond to carbon (C) and oxygen (O), which are characteristic of carbon materials (Figure 2a,e) [24]. In the EDX spectrum of HCK, a peak corresponding to potassium (K) was also observed, indicating the successful modification and incorporation of K ions onto the surface of the hydrochar. Similar conclusions were reported by Petrović et al. [15] upon KOH binding onto hydrochar and Simić et al. [9] during alkali modification of corn silk. After the adsorption of organophosphorus pesticides using HCK (Figure 2b,c), there was a simultaneous decrease in O and K, suggesting the involvement of surface oxygenated functional groups and ion exchange in binding interactions with the pesticides [15]. Appearance of new peaks corresponding to chlorine (Cl), phosphorus (P), sulfur (S), and nitrogen (N) and an increase in C confirms the successful adsorption of CHP and AZM, as these elements are inherent components of its molecular structure. During the removal of the selected pesticides utilizing PHC (Figure 2f,g), a reduction in both C and O content was observed, accompanied by the appearance of new peaks (Cl, P, S, and N). These changes suggest that despite the low surface polarity of PHC, the pesticide is likely adsorbed through surface interaction (π-π) and partial surface complexation with residual functional groups [25]. The appearance of new peaks additionally confirms successful pesticide adsorption. The applied modification significantly alters the surface chemistry of the hydrochars, particularly the distribution of oxygen-containing groups, thereby influencing the adsorption mechanisms involved in pesticide removal.

Along with RB dye adsorption (Figure 2d,h), an increase in the C content and a decrease in the O content were observed for both tested materials. Additionally, new peaks (N and Cl) were detected in the EDX spectra, as previously explained. The similar adsorption trend of cationic dye RB observed for both PHC and HCK suggests that surface morphology plays a less dominant role in this case. Instead, adsorption is likely governed by chemical interaction between positively charged RB molecules and negatively charged oxygen-containing functional groups of HCK and PHC. Similar observations were reported by You et al. during the investigation of dye removal using modified rice husk powder [26].

**Figure 3:**
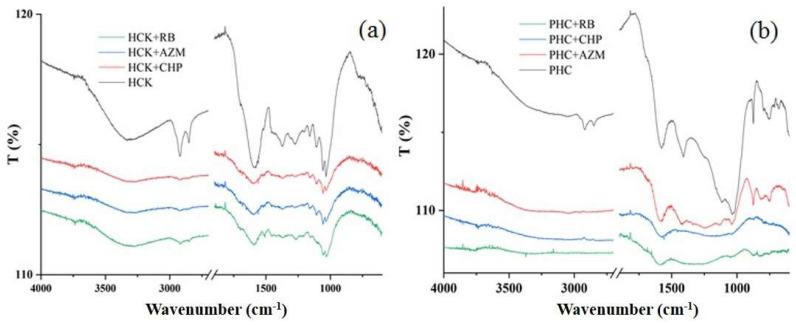
FTIR spectra of HCK (**a**) and PHC (**b**) before and after adsorption of CHP, AZM, and RB.

The FTIR spectra of the two hydrochar samples, HCK and PHC, exhibit distinct spectral features that reflect the chemical transformations induced by their modification treatments (Figure 3a,b). The FTIR spectra of HCK display a broad band around 3323 cm^−1^, as well as bands at 1058 cm^−1^ and 1033 cm^−1^, corresponding respectively to O-H stretching vibrations of hydroxyl groups and C-O stretching vibrations of primary and/or alkyl-substituted ethers [5,17]. This indicates a higher density of hydroxyl functionalities introduced or exposed through alkaline modification, which significantly enhances the surface polarity and availability of adsorption sites. The presence of pronounced methylene (-CH_2_-) stretching vibrations at 2919 and 2849 cm^−1^ further suggests the retention or formation of aliphatic structures, consistent with a less disrupted carbonaceous framework [5,14]. Additionally, the HCK spectrum shows a band at 1112 cm^−1^ attributed to C-O stretching vibrations of alcohols, ethers, and/or phenolic groups, aromatic C=C and C-H banding vibrations at 1594 cm^−1^ and around 800 cm^−1^, and a band at 1374 cm^−1^ assigned to symmetric COO^−^ stretching of carboxylates [27,28,29]. Conversely, PHC exhibits markedly lower intensities of hydroxyl bands (at 3356 cm^−1^) and methylene-stretching vibrations (2981 and 2851 cm^−1^) compared to HCK, suggesting the partial removal of oxygen-containing functional groups through thermal decomposition during pyrolysis. Furthermore, the reduced intensity of the aromatic C=C stretching band near 1600 cm^−1^ indicates structural rearrangements associated with carbonization processes that reduce aromatic unsaturation. In contrast, the increased intensity of bands assigned to aromatic C-H out-of-plane deformations in the 800-600 cm^−1^ region become more pronounced after pyrolysis, probably due to enhanced structural aromaticity [15]. Notably, PHC still shows a band around 1405 cm^−1^, corresponding to COO^−^ stretching, and a band near 1033 cm^−1^ associated with C-O stretching, similar to HCK, indicating that some oxygenated functionalities are retained even after pyrolysis.

After adsorption of pesticides and RB, the FTIR spectra of HCK and PHC showed decreases in the intensity of hydroxyl, methylene, and carboxylate bands, along with shifts toward lower wavenumbers, indicating interactions between surface functional groups and the adsorbates (Figure 3a,b). These changes indicate that surface functional groups were altered during the adsorption process [28]. In the case of HCK, pronounced variations were observed across a wide range of functional groups, indicating stronger and more diverse interactions with the adsorbates. Conversely, PHC exhibited more localized spectral shifts, particularly in regions associated with methylene (-CH_2_-) and aromatic groups, suggesting selective adsorption primarily governed by hydrophobic effects and π-π binding interactions [4,22]. Upon AZM adsorption, a new band at 1256 cm^−1^, corresponding to P=O stretching vibrations of AZM, appears in the PHC spectrum, indicating potential interaction of AZM with the hydrochar surface [30]. Additionally, the reduced intensity of peaks within the 1030–1110 cm^−1^ region may indicate interactions between the phosphate groups of both AZM and CHP and the surface hydroxyl/carboxyl functionalities, consistent with previously reported phosphate–carbon interactions [31]. Furthermore, after RB adsorption on PHC, the band originally observed at 1405 cm^−1^ (COO^−^ stretching vibrations), and the 1030–1110 cm^−1^ region shifts slightly to lower wavenumbers and exhibits a decrease in intensity. This behavior indicates that the carboxylate groups on the hydrochar surface are involved in interactions with RB molecules, likely through electrostatic attraction and hydrogen bonding. These changes are consistent with established adsorption mechanisms of dyes on carbon-based materials, where functional groups on the adsorbent surface play a key role in pollutant binding [31]. These results align with results of EDX analysis and previously reported findings on the adsorption of organic pollutants onto carbonaceous materials, where multiple adsorption pathways are typically involved [21,28].

To clarify the role of pH in the adsorption process, the pH_PZC_ values of the materials were determined (Figure S1). The alkaline-treated HCK showed a pH_PZC_ of 6.02, while PHC had a slightly lower value of 5.40. Since the adsorption experiments were carried out at pH 6, HCK operated very close to its pH_PZC_ and therefore had an almost neutral to mildly negative surface, whereas PHC was clearly negatively charged [5,32]. This difference in surface charge suggests potential adsorption tendencies. The more negatively charged PHC surface supports the adsorption of neutral organophosphorus pesticides (CHP and AZM), whose removal depends mainly on non-electrostatic interactions such as hydrophobic forces and π-π interactions [5,7]. In contrast, RB is predominantly cationic at pH 6; thus, its stronger affinity for HCK can be attributed to the nearly neutral surface of this material, which minimizes repulsion and allows other interactions (e.g., π-π binding and hydrogen bonding) to contribute more effectively. Moreover, Figure S2 presents the nitrogen adsorption isotherms for HCK and PHC samples, measured as the amount of N_2_ adsorbed at −196 °C. The specific surface area (S_BET_) for each sample, calculated using the BET equation, was 8 m^2^ g^−1^ for HCK and 35 m^2^ g^−1^ for PHC. Both isotherms are of type II, which is characteristic of non-porous or macroporous adsorbents [33]. According to the BET analysis, it can be concluded that HCK has a very low surface area, while that of PHC is considerably higher. This contrast reflects the distinct impact of alkaline activation versus secondary pyrolysis on hydrochar morphology and porosity. These observations align with the widely established view that structural disorder in carbonaceous materials, expressed through fragmented aromatic domains, heterogeneous surface sites, and oxygen-enriched defect regions, enhances adsorption performance by increasing both the density and accessibility of energetically active binding sites [34]. The observed trends are supported by the PSD analysis (Figure S3). HCK exhibited a broad PSD spanning the full measured range, with the highest intensities in the 50–100 and 100–200 µm fractions. This is consistent with hydrochars that largely preserve the macroscopic structure of the precursor biomass after alkaline treatment, as commonly reported for HTC-derived materials with limited mechanical disintegration during chemical activation [35]. KOH activation introduces oxygen-containing functional groups and surface defects but does not substantially fragment the material, resulting in larger, less porous particles where adsorption predominantly occurs on chemically active surface sites [36]. In contrast, PHC exhibited a narrower PSD, with the most prominent peaks at <25 and 25–50 µm, while particles larger than 75 µm were present at much lower intensities. Secondary pyrolysis following hydrothermal carbonization is known to thermally weaken particle integrity, causing fragmentation and generating smaller, more uniform particles with increased accessible surface area [37]. This structural transformation directly supports the higher BET surface area of PHC and contributes to more efficient mass transfer and faster diffusion during adsorption. The smaller particle size and enhanced porosity of PHC thus facilitate the adsorption of neutral pesticides through hydrophobic and π-π interactions. Consistent with this interpretation, Liu et al. recently demonstrated for nanoporous carbons that reduced graphene-like domain size and increased structural disorder lead to enhanced functional performance, reinforcing our view that PHC’s more fragmented morphology and higher accessible surface facilitate adsorption [38]. Such morphology–performance relationships are further in line with the broader principles highlighted by Manjunath et al., who emphasize that activation route, particle fragmentation, and the availability of oxygenated surface sites are key determinants of adsorption efficiency in low-cost biomass-derived adsorbents [39].

The combined characterization results highlight the complementary roles of surface chemistry, porosity, and surface charge in adsorption potential. HCK, despite its low surface area (8 m^2^ g^−1^), possesses a high density of oxygen-containing functional groups introduced by KOH treatment (FTIR), and a nearly neutral surface at pH 6 (pH_PZC_ = 6.02), which facilitates the removal of cationic contaminants via π-π interactions and hydrogen bonding, with only modest contributions from electrostatic forces. In contrast, PHC exhibits a higher surface area (35 m^2^ g^−1^) and a more negatively charged surface at the same pH (pH_PZC_ = 5.40), favoring the adsorption of neutral organophosphorus pesticides primarily through hydrophobic effects and π-π interactions, while electrostatic interactions play a negligible role. These observations indicate that although pH_PZC_ provides insight into potential charge-related interactions, the overall adsorption behavior is largely governed by the synergy between surface functionalization and accessible porosity. These findings align with those of Tasić et al., highlighting that adsorption is mainly driven by surface chemistry and porosity, with limited influence of electrostatic interactions at pH 6 [6].

The structural integrity of adsorbents is important for their effectiveness, so we also tested the ultrasonic stability of the hydrochars to see how well they resist mechanical stress and leaching. Both HCK and PHC samples remained stable after being exposed to 35 kHz sonication in water for 30 min at 25 °C. A minor mass loss of approximately 5.8% was observed for the HCK sample, accompanied by slight turbidity in the dispersion medium, probably due to the release of loosely attached surface residues from the alkaline treatment. In contrast, the PHC sample showed almost no mass variation and retained full dispersion clarity, suggesting higher resistance to ultrasonic-induced degradation. Turbidimetry at 650 nm further confirmed these observations, showing a minor absorbance change for PHC (from 0.005 to 0.009) and a pronounced increase for HCK (from 0.050 to 0.366) after sonication, consistent with the observed mass loss. This mechanical stability is particularly relevant for potential applications in water treatment, where preservation of material structure under dynamic conditions is essential. Reports on the ultrasonic stability of hydrochars are scarce, highlighting the relevance of these findings as a reference point for assessing the mechanical integrity of bio-based adsorbents under dynamic aqueous conditions. The different results for HCK and PHC also show how post-treatment changes can affect hydrochar durability.

### 2.2. Adsorption Kinetics

**Figure 4:**
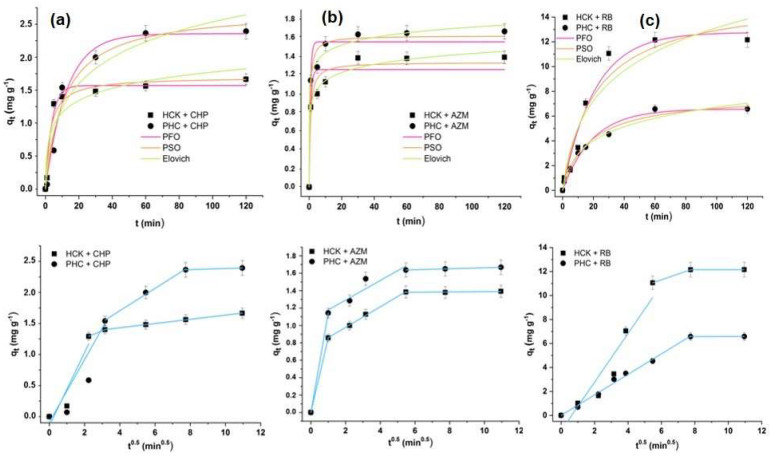
Kinetic adsorption curves (mean ± SD, n = 3) of (**a**) HCK and PHC for CHP, (**b**) HCK and PHC for AZM, and (**c**) HCK and PHC for RB (1 mg mL^−1^, 5 × 10^−5^ mol dm^−3^, pH = 6).

Rapid initial adsorption was observed for all pollutants, with equilibrium reached within 20 min for CHP, 10 min for AZM, and 60 min for RB (Figure 4a–c). The fast initial adsorption reflects the abundance of accessible active sites on HCK and PHC surfaces [5]. To investigate the adsorption mechanisms, kinetic data were analyzed using non-linear pseudo-first-order (PFO), pseudo-second-order (PSO), Elovich (EKM), and intraparticle diffusion (IPD) models (Table 1, Figure 4). This multi-model approach provides mechanistic insights rather than merely identifying the statistically best fit.

Based on the R^2^ and χ^2^ values (Table 1), the PFO model better describes RB and CHP adsorption on HCK, whereas the PSO model provides a better fit for AZM adsorption on HCK and for the removal of all three pollutants using PHC. Similar trends have been reported for the adsorption of RB on citric acid-modified banana peel and CHP adsorption on spent coffee grounds [40]. However, although the PSO model is sometimes associated with stronger adsorbent-adsorbate interactions, kinetic modeling alone cannot be used to determine the adsorption mechanism. As emphasized by Lima et al. [41], PFO and PSO models describe the mathematical form of the adsorption rate rather than the underlying adsorption mechanism.

Additional insight into the adsorption dynamics is provided by the EKM analysis [42]. For RB and AZM adsorption on both materials, the α parameter (initial adsorption rate) is significantly higher than β (desorption rate), indicating that adsorption predominates over desorption. In contrast, for CHP, β exceeds α, suggesting weaker adsorbent–adsorbate interactions and a greater contribution of reversible adsorption.

The IDP model indicates that the adsorption process proceeds through diffusion-controlled stages. For CHP on HCK, AZM on HCK, and RB on PHC, two phases are observed: an initial stage governed by external (film) diffusion, followed by intraparticle diffusion within the adsorbent pores. In contrast, CHP on PHC, AZM on PHC, and RB on HCK exhibit three phases, where the two diffusion steps are followed by a final equilibrium stage characterized by a markedly slower adsorption rate (Figure 4a–c). Similarly, Yao et al. showed that the removal process of pesticides on KOH-activated crayfish shell biochar occurs in two phases, while the research of Milanković et al. proved that the adsorption of Malathion removal on spent coffee grounds takes place in three phases [4,43]. In addition, the decrease in k_id_ values after each breakpoint (Table 1) indicates a progressive reduction in the diffusion rate as adsorption proceeds and active sites become occupied, while the increase in C values highlights the contribution of boundary layer resistance to the overall adsorption process.

In addition to differences in adsorption kinetics for CHP, AZM, and RB, the variations in adsorption capacity of HCK and PHC are reflected in the q_e_ values (Table 1) and the fit to pseudo-first-order (PFO) or pseudo-second-order (PSO) models. HCK exhibits a higher capacity for cationic dye RB, consistent with its nearly neutral surface (pH_PZC_ = 6.02) and abundant hydroxyl/carboxyl functional groups from KOH activation, and is better described by the PFO model, indicating rapid initial uptake and slower equilibrium. Conversely, PHC shows higher capacity for neutral organophosphorus pesticides (CHP, AZM), with larger BET surface area, smaller particle size, and higher aromaticity due to secondary pyrolysis, and fits the PSO model, reflecting adsorption rates dependent on both adsorbent and adsorbate concentrations. Together, q_e_ values and kinetic modeling demonstrate that adsorption performance arises from the synergy between surface chemistry, porosity, particle size, and surface charge, providing a comprehensive explanation for the observed differences between HCK and PHC.

### 2.3. Adsorption Isotherms

The adsorption experiments were conducted at temperatures of 22, 30, and 35 °C, with pollutant concentrations ranging from 1∙10^−5^ mol dm^−3^ to 2.5∙10^−4^ mol dm^−3^ for RB dye and from 1∙10^−4^ mol dm^−3^ to 1∙10^−6^ mol dm^−3^ for pesticides. In contrast, the adsorbent concentration was kept constant at 1 mg mL^−1^. The collected data were analyzed using various non-linear isotherm models, as summarized in Table 2, Table 3 and Table 4, including the Freundlich, Langmuir, Temkin, and Dubinin–Radushkevich models (Figure S4). All experimental data points in the isotherm plots include error bars representing standard deviations, while each fitted model is shown in a distinct color and clearly identified in the legend for visual clarity.

These isotherm results indicate that the HCK and PHC adsorbents exhibit good adsorption performance for the tested pollutants, while the adsorption capacities and the equilibrium behavior are slightly different from each other (Figure S4). The Freundlich isotherm model was employed to determine the parameter n, which provides insight into the surface heterogeneity of the adsorbents [14,44]. From the data presented in Table 2, Table 3 and Table 4, it is evident that the value of the n parameter for RB (HCK, PHC) and CHP (HCK, PHC) increases with temperature. This suggests that the adsorption sites on the tested adsorbents become more available for dye and pesticide adsorption at higher temperatures, probably due to the enhanced reactivity of the sorbent surface [45]. In contrast to this trend, for AZM (HCK, PHC), the n values decrease as the temperature rises. Importantly, all values of n greater than 1 indicate that the adsorption process is favorable for the tested dyes and pesticides [14,44].

Based on the parameters presented in Table 2, Table 3 and Table 4, the adsorption data are generally best described by the Langmuir isotherm, suggesting relatively uniform adsorption sites and monolayer coverage of the contaminants on the adsorbent surface [46]. However, the R^2^ values show that the Freundlich, Temkin, and Dubinin–Radushkevich models can also provide a reasonable fit, with the best-fitting model depending on the specific adsorbent–pollutant combination. Specifically, for PHC at 22 °C, the Freundlich model better represents the adsorption of all three pollutants, whereas at higher temperatures, the Langmuir model provides a superior fit. This transition reflects a temperature-dependent change in adsorption behavior, indicating that surface site homogeneity and adsorption energetics may vary with temperature.

Temperature significantly influenced adsorption performance. For HCK, increasing the temperature from 22 to 35 °C progressively enhanced the adsorption capacity for all pollutants, with q_max_ values rising from 2.34 to 3.98 mg g^−1^ for CHP, from 0.94 to 1.39 mg g^−1^ for AZM, and from 132 to 751 mg g^−1^ for RB. This increase is attributed to higher molecular mobility and more frequent collisions between pollutant molecules and active adsorption sites at elevated temperatures, facilitating adsorption. Similar effects have been reported for RB on carbon-based materials [47]. In contrast, PHC displayed pollutant-specific temperature dependence: CHP adsorption was highest at 22 °C (30.1 mg g^−1^), AZM adsorption peaked at 30 °C, and RB removal reached a maximum of 616.0 mg g^−1^ at 35 °C. These differences indicate that the temperature dependence of adsorption is determined by the interplay between the adsorbent’s structural features, such as surface area and functional groups, and the chemical characteristics of the pollutant, including size, polarity, and reactivity.

The Dubinin–Radushkevich model yielded low mean adsorption energy values (E << 8 kJ mol^−1^), suggesting that physisorption dominates the adsorption process, likely driven by weak intermolecular interactions such as van der Waals forces [48]. Complementarily, the Temkin model indicates that the heat of adsorption (b_T_) decreases with increasing temperature, implying that as more adsorption sites become occupied, the interaction energy between the adsorbent and pollutants diminishes, resulting in reduced adsorption heat at higher surface coverage [43]. Overall, these models indicate that adsorption is mainly governed by physical interactions and that increasing surface coverage limits the number of available active sites, resulting in a decrease in adsorption energy.

The maximum adsorption capacities calculated from the Langmuir model were 3.98, 1.39, and 751 mg g^−1^ for CHP, AZM, and RB on HCK, respectively. In contrast, PHC exhibited higher capacities for the pesticides, with 30.1 mg g^−1^ for CHP and 9.15 mg g^−1^ for AZM, while RB adsorption reached 616.0 mg g^−1^. These differences can be attributed to the distinct structural and chemical properties of the hydrochars. PHC possesses a higher BET surface area and a greater density of polar functional groups, such as carboxyl and phenolic moieties, which enhance the adsorption of organophosphorus pesticides. Conversely, HCK is enriched in oxygen-containing functional groups that preferentially interact with cationic dyes via hydrogen bonding and electrostatic attractions [49]. Similar selectivity trends have been observed for the adsorption of organic pollutants on other biomass-derived carbon materials [18].

Overall, the adsorption performance of the grape pomace-derived hydrochars in this study was equal to or exceeded that of previously reported hydrochars, biochars, and other carbon-based materials for RB and pesticide removal (Table 5) [50,51,52]. This demonstrates that hydrochars produced via pyrolysis and alkaline modification possess a high affinity for the investigated contaminants, highlighting their strong potential for practical applications in environmental remediation.

These observations regarding adsorption capacity, selectivity, and temperature dependence are further supported by structural and spectroscopic analyses, which provide direct evidence of the interactions between the pollutants and the hydrochar surfaces. To critically evaluate the high adsorption capacities observed for RB, SEM images (Figure 1d,h) show no evidence of precipitation or aggregation. EDX and FTIR analyses indicate that RB interacts with HCK mainly through electrostatic attraction, hydrogen bonding, and dipole–dipole interactions with surface hydroxyl and carboxyl groups, while PHC binds RB via π-π binding and hydrophobic interactions. Despite HCK’s low BET surface area, accessible functional groups facilitate efficient adsorption. Favorable surface chemistry and strong adsorbent–adsorbate interactions, rather than multilayer adsorption or experimental artifacts, consistent with the Langmuir isotherm and monolayer adsorption, thus explain the high q_max_ values. These observations are further corroborated by the thermodynamic analysis presented in the following section, which confirms that adsorption processes are spontaneous and driven by favorable enthalpic and/or entropic contributions.

This study introduces a significant innovation by successfully removing AZM for the first time using hydrochars. Furthermore, it is the first study to systematically test pyro-hydrochars within this framework for the removal of specific pollutants, providing novel insights into the efficacy of hydrochars as adsorbents for these contaminants.

### 2.4. Thermodynamic Study

Thermodynamic parameters were determined to better understand the affinity between the tested pollutants and hydrochar surfaces (Table S1), following best practices for adsorption evaluation [57]. Van’t Hoff plots for CHP, AZM, and RB are shown in Figure S5, with the calculated ΔH°, ΔS°, and ΔG° values presented in Table 6.

For CHP and RB, adsorption onto both hydrochars is endothermic (ΔH° > 0) and accompanied by an increase in entropy (ΔS° > 0), indicating spontaneous processes (ΔG° < 0) driven by enhanced molecular mobility and increased disorder at the solid-solution interface [7,58]. The notably higher ΔS° values for RB compared to CHP suggest stronger interactions of RB with the hydrochar surfaces, likely due to electrostatic attraction and hydrogen bonding with oxygen-containing functional groups. In contrast, AZM adsorption is primarily enthalpy-driven, particularly on PHC (ΔH° < 0), and is associated with smaller or negative ΔS°, indicating that specific binding interactions, such as hydrogen bonding or π-π binding, dominate over entropic contributions. These findings demonstrate that the temperature dependence and spontaneity of adsorption are governed by the combined effects of adsorbent structure, surface chemistry, and the molecular properties of the pollutants, including polarity, size, and functional groups.

The low adsorption energies from the Dubinin–Radushkevich model (E << 8 kJ mol^−1^) indicate that adsorption is largely governed by physical interactions, such as van der Waals forces and hydrogen bonding, with potential contributions from other interactions depending on the hydrochar and pollutant [48]. The Temkin model shows that the heat of adsorption (b_T_) decreases with increasing surface coverage, reflecting lower average interaction energies as sites become occupied [43].

Integrating thermodynamic, kinetic, and surface chemistry evidence reveals two distinct adsorption regimes for the hydrochars. HCK, enriched in hydroxyl and carboxyl groups, facilitates rapid, entropy-driven adsorption of polar and cationic pollutants through electrostatic attraction and hydrogen-bonding interactions, in agreement with pseudo-first-order kinetics [15,24]. In contrast, PHC, with its rigid aromatic framework and reactive surface functional groups, exhibits slower, enthalpy-driven adsorption. This process is primarily governed by specific interactions, including hydrogen bonding, π-π and coordinative binding, consistent with pseudo-second-order kinetics [4,59]. This comparison highlights how differences in surface chemistry and structural features dictate both the rate and the nature of adsorbent–pollutant interactions.

To validate and interpret the experimentally observed adsorption behavior, MEP maps were generated for CHP, RB, and AZM, as well as for representative molecular fragments of the two hydrochar-based adsorbents: HCK and PHC. MEP/ESP analysis is widely used to identify reactive centers and predict adsorption orientations on carbon-based materials, including biochar and hydrochar analogues [60].

**Figure 5:**
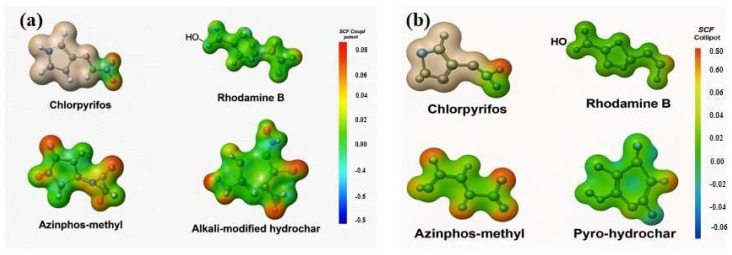
Molecular electrostatic potential (MEP) maps of CHP, AZM, and RB and representative surface fragments of HCK (**a**) and PHC (**b**).

MEP analysis (Figure 5) provides further molecular-level support. HCK surfaces exhibit electron-deficient zones conducive to electrostatic and hydrogen-bonding interactions with electron-rich moieties of CHP, AZM, and RB [60,61]. PHC presents extended π-systems that facilitate π-π and hydrophobic interactions, particularly relevant for AZM and CHP, whose adsorption is predominantly enthalpy-driven and temperature-sensitive [59,61]. Overall, MEP results corroborate the thermodynamic and FTIR analyses, explaining the differential affinities of pollutants for HCK and PHC and confirming that adsorption is governed by multiple concurrent interactions rather than a single mechanism.

Integration of kinetic, isotherm, thermodynamic, and MEP analyses provides a coherent mechanistic understanding of pollutant adsorption by HCK and PHC. On HCK, adsorption is rapid and entropy-driven, dominated by physical interactions such as electrostatic attractions and hydrogen bonding. In contrast, PHC exhibits slower, enthalpy-driven adsorption, primarily mediated by specific interactions such as hydrogen bonding. These findings demonstrate that adsorption behavior is intrinsically dependent on the adsorbent’s surface chemistry, functional groups, porosity, and the molecular characteristics of the pollutant, with MEP analysis confirming the predominant interaction regimes at the molecular level.

### 2.5. Cost Analysis

The laboratory-scale production cost of the synthesized hydrochars was estimated based on electricity, chemical reagents, and filter papers (Table S2). For HCK, hydrothermal carbonization at 220 °C for 1 h, followed by KOH modification and filtration, resulted in ≈32.8 USD kg^−1^. The PHC sample, which included an additional pyrolysis step but no KOH, cost ≈13.3 USD kg^−1^. The calculated production costs account for the obtained hydrochar yields (79.0% for HCK, 64.90% for PHC).

For comparison, Cassia fistula-derived activated carbon (CFPAC) was reported at ≈5.46 USD kg^−1^ lab scale [62] and waste cherry kernel-derived AC (CScPA) at ≈41.9 USD kg^−1^. Darco^®^ CAC, a commercially available activated carbon, is priced at ≈271 USD kg^−1^ and serves as a market reference [63].

Thus, HCK is more expensive than PHC due to chemical activation, but both hydrochars demonstrate that HTC is a cost-effective method for producing functional carbon materials compared to commercial alternatives.

## 3. Materials and Methods

### 3.1. Chemicals

All of the chemicals and reagents used in the present study were of analytical grade. The primary standard solution of RB and pesticides was prepared by dissolving the weighed amount of chemicals (Sigma Aldrich, Søborg, Denmark) in ultra-distilled water. Solutions of various concentrations used in experiments were prepared by diluting the primary stock solution with ultra-distilled water. For modification treatment, 2 M potassium hydroxide (KOH) solution was prepared from a solid chemical (Sigma-Aldrich, Hamburg, Germany). As the mobile phase was used, acetonitrile (J.T. Baker, Phillipsburg, NJ, USA) was used.

### 3.2. Preparation and Modification of GP Hydrochar

The GP was air-dried, ground, and sieved to achieve homogeneity with a particle size of less than 0.5 mm. The specific preparation of GP hydrochar through hydrothermal carbonization (HTC) treatment at 220 °C for one hour has been discussed in detail in our previous studies [15]. For the carbonization process, 15 g of GP was mixed with 150 mL of distilled water (1:10 ratio) and subjected to hydrothermal carbonization in a laboratory autoclave (Carl Roth, model II, 250 mL) at 220 °C for 1 h. The produced hydrochar (HGP) underwent modification through two distinct methods. The first method involved alkali activation, where 1 g of HGP was stirred with 100 mL of 2 M KOH solution for one hour at room temperature (25 ± 0.5 °C) [15]. The activated hydrochar, labeled as HCK, was then filtered and rinsed with distilled water, and its pH was adjusted to neutral utilizing 0.1 M HNO3/KOH. Afterward, it was filtered again and dried overnight at 105 °C.

In the second modification process, pyrolysis of HGP (2 g) was conducted in a quartz crucible at 400 °C, with a heating rate of 10 °C min^−1^, under an inert atmosphere (N_2_ flow at 80 mL min^−1^). This process, completed within one hour using a furnace (Nabertherm 30–3000 °C, Lilienthal, Germany), resulted in a pyro-hydrochar labeled as PHC.

### 3.3. Characterization of Obtained Hydrochars Before and upon Adsorption

The SEM analysis was performed to elucidate the surface morphology and structural characteristics of HCK and PHC before and after the adsorption of pesticides and dye. The surface morphologies of both materials, before and after adsorption, were examined using a SEM-EDX Microscope model JSM-700 1F (JEOL Inc., Peabody, United States of America). Prior to recording, all samples were gold-coated and mounted on adhesive carbon discs. SEM images were recorded at a magnification of 500× using an accelerating voltage of 20.0 kV. For elemental analysis, representative surface regions of multiple hydrochar particles (n ≥ 3) were examined using the EDX functionality of the SEM instrument. The spectra were collected from selected areas corresponding to those imaged in SEM, and the reported elemental compositions represent the average of these measurements. Particle size distribution of the hydrochar samples was analyzed from SEM images using ImageJ (version 1.53k, National Institutes of Health, Bethesda, MD, USA), with measurements performed on 250 individual particles [35]. Spectroscopic analysis was performed using a Thermo Scientific Nicolet iS50 FT-IR spectrometer (Thermo Scientific, Massachusetts, United States of America) equipped with an ATR accessory to examine the functional groups and molecular vibrations of the hydrochars before and after pollutant adsorption. The measurements were conducted in ATR mode with 32 scans over the spectral range of 4000–400 cm^−1^. This approach provided valuable information on the chemical composition and the role of surface functional groups involved in the adsorption. The point of zero charge (pH_PZC_) of the samples before and after modification was determined following the procedure described by Milonjic et al. [64]. According to this method, 0.01 g of the selected material was added to a 0.01 M KNO_3_ solution whose initial pH (pH_i_) had been adjusted to values between 2.0 and 12.0 using 0.01 M HNO_3_ or 0.01 M KOH. After a 24 h equilibration period, the final pH (pH_f_) was determined using a digital pH meter (AMT20, Amtast, Amstat Industries, Zurich, Switzerland), and a plot of pH_f_ versus pH_i_ was constructed to determine the pH_PZC_. The specific surface areas (S_BET_) of the materials were analyzed via low-temperature nitrogen adsorption according to the Brunauer–Emmet–Teller (BET) method [65] using a Surfer porosimeter (Thermo Fisher Scientific, Thermo Scientific, Massachusetts, United States of America). Prior to analysis, the samples underwent degassing at 100 °C for 6 h under vacuum. For ultrasonic stability, 50 mg of hydrochar was dispersed in 50 mL of distilled water and subjected to sonication at 35 kHz for 30 min at 25 °C. Stability was evaluated by measuring mass loss and visually inspecting dispersion. Additionally, the turbidity of the suspensions was monitored using ultraviolet–visible spectrometer (UV-Vis) (Jena Specol 1300, Jena, Germany) measurements at a wavelength of 650 nm before and after sonication [66]. This process was designed to assess the structural integrity and dispersion behavior of the materials under mechanical stress. The cost analysis provides an overview of the economic aspects associated with the laboratory-scale production of the synthesized hydrochars. The production cost was estimated considering electricity consumption, chemical reagents, and filtration materials used during synthesis. The costs were calculated based on the quantities consumed and normalized to the mass of the produced material (USD kg^−1^), taking into account the hydrochar yield determined from the mass difference before and after the corresponding modification step. The approach follows previously reported methodologies for biomass-derived activated carbons [63].

### 3.4. Adsorption Experiments

Two organophosphorus pesticides, CHP and AZM, were selected as representative pesticide pollutants for adsorption studies, along with the model dye RB, to evaluate the adsorption potential of HCK and PHC under controlled conditions. Adsorbent dispersions (1 mg mL^−1^) were incubated with pesticide solutions (5 × 10^−1^ mol dm^−1^) for 1 h. The residual pesticide concentration was quantified via Ultra-Performance Liquid Chromatography (UPLC), and adsorption efficiency was calculated as the percentage removed relative to the initial concentration. UPLC analysis was performed using a Waters ACQUITY system with a PDA detector (Empower 3 software), equipped with an ACQUITY UPLC™ BEH C18 column (1.7 μm, 100 × 2.1 mm) under isocratic elution (30% water-acetonitrile: 70% acetonitrile) at 0.20 mL min^−1^, with detection at 200 nm and an injection volume of 5 μL.

Working dispersions were prepared by mixing the adsorbents (1 mg mL^−1^) with pollutant stock solutions. To evaluate the efficiency of different activation approaches, the adsorption performance of the carbonized materials was examined under conditions representative of natural aquatic environments (pH 6, 22 °C, and pollutant levels typical for wastewater).

Kinetic experiments were performed at 22 °C and pH 6, over contact times ranging from 1 to 120 min, using initial pollutant concentrations of 5 × 10^−5^ mol dm^−3^ (corresponding to 23.95 mg L^−1^ for RB, 15.87 mg L^−1^ for AZM, and 17.53 mg L^−1^ for CHP). This concentration was selected to be higher than that employed during the preliminary screening stage to enable a clearer evaluation of kinetic parameters, since the lower concentration was almost completely adsorbed within a very short contact time. Adsorption isotherm experiments were conducted under the same pH conditions (pH 6) at 22, 30, and 35 °C, employing initial concentration ranges of 1 × 10^−6^ to 1 × 10^−4^ mol dm^−3^ for CHP and AZM and 1 × 10^−5^ to 2.5 × 10^−4^ mol dm^−3^ for RB, with an equilibrium contact time of 120 min. The thermodynamic experiments were conducted at 22, 30, and 35 °C, using a pollutant concentration of 5 × 10^−5^ mol dm^−3^, pH 6, and a contact time of 120 min, in order to evaluate the temperature dependence of adsorption under environmentally relevant conditions. Following incubation, the suspensions were centrifuged at 14,500 rpm, and the supernatants were analyzed. Pesticide concentrations were quantified by means of UPLC, while RB was analyzed by means of UV-Vis (Perkin Elmer Lambda 35) at 554 nm. Control experiments without an adsorbent were carried out under identical conditions. All adsorption measurements were performed in triplicate, and the results are expressed as mean ± standard deviation to ensure reproducibility.

The amounts of removed dye and pesticides utilizing both HCK and PHC at equilibrium *q_eq_* were calculated by following equation:

(1) $$q_{eq}=\frac{C_{0}-C_{eq}}{m}\times V$$

where *V* represents the volume of the solution (L), *m* is the amount of adsorbent (g), and *C*_0_ and *C_eq_* are the initial and equilibrium concentrations of the selected pollutant (mg L^−1^), respectively.

To study adsorption kinetics and isotherm models, both linear and non-linear fitting methods were utilized using Origin 9.0 software.

To support the interpretation of the adsorption mechanisms and provide a molecular-level understanding of the interactions between pollutants and the hydrochar-based adsorbents, molecular electrostatic potential (MEP) map analysis was conducted. These calculations aimed to provide insight into the intermolecular forces governing the adsorption of CHP, RB, and AZM, as well as for representative structural fragments of HCK and PHC.

## 4. Conclusions

This study explores the novel application of grape pomace hydrochars as potential adsorbents for the removal of specific organophosphorus pesticides (CHP and AZM) and the synthetic dye RB. The hydrochars, modified through pyrolysis and alkaline treatment, exhibited different structural characteristics that significantly affect their adsorption performance. Between the two treatments, pyrolized hydrochar (PHC) showed superior performance in pesticide removal, with maximum adsorption capacities of 30.10 mg g^−1^ for CHP and 9.15 mg g^−1^ for AZM. In contrast, alkaline-modified hydrochar (HCK) demonstrated a significantly higher capacity (751.0 mg g^−1^) for RB removal compared to PHC, underscoring its potential as a highly efficient dye adsorbent. Physicochemical characterization through SEM, FTIR, BET, and pH_PZC_ revealed differences in surface morphology, functional groups, porosity, and surface charge, highlighting the complementary roles of surface chemistry and porosity in adsorption performance. Adsorption was primarily driven by π-π interactions, hydrogen bonding, and electrostatic and dipole-dipole forces, with hydrophobic interactions further contributing to selectivity. The adsorption kinetics followed distinct models, indicating different interaction mechanisms between HCK and PHC with the pollutants. Thermodynamic studies, alongside MEP analysis, confirmed that the adsorption processes were spontaneous and thermodynamically favorable under the tested conditions. Moreover, ultrasonic testing confirmed that post-treatment influences hydrochar stability, with PHC showing superior structural integrity. This supports its suitability for use in dynamic aqueous systems. Despite their simplicity, KOH activation and pyrolysis significantly improved the adsorptive properties of the hydrochars. Overall, the results indicate that PHC is more suitable for the removal of organophosphorus pesticides, whereas HCK is particularly effective for the adsorption of cationic dyes such as RB. These findings suggest that grape pomace-derived hydrochars offer a promising, sustainable solution for the targeted removal of specific contaminants, contributing to both effective wastewater treatment and the valorization of agricultural waste as cost-effective materials.

## Figures and Tables

**Table 1:** Kinetic parameters for RB, CHP, and AZM adsorption using HCK and PHC.

M.	HCK	PHC
Pollutant	PFO	
RB		
q_e_ (mg g^−1^)	12.76	6.57
k_1_ (min^−1^)	0.048	0.052
χ^2^	0.94	0.14
Adj. R^2^	0.9650	0.9773
BIC	1.38	−13.89
CHP		
q_e_ (mg g^−1^)	1.51	2.29
k_1_ (min^−1^)	0.32	0.074
χ^2^	0.049	0.069
Adj. R^2^	0.8339	0.9215
BIC	5.84	−16.57
AZM		
q_e_ (mg g^−1^)	1.26	1.55
k_1_ (min^−1^)	1.10	1.30
χ^2^	0.033	0.025
Adj. R^2^	0.3866	0.4832
BIC	9.45	−13.89
	PSO	
RB		
q_e_ (mg g^−1^)	15.64	7.75
k_2_ (mg min^−1^ g^−1^)	0.003	0.007
χ^2^	1.64	0.099
Adj. R^2^	0.9389	0.9837
BIC	−16.84	−14.82
CHP		
q_e_ (mg g^−1^)	1.63	2.67
k_2_ (mg min^−1^ g^−1^)	0.250	0.031
χ^2^	0.068	0.057
Adj. R^2^	0.7723	0.9357
BIC	−14.95	−16.02
AZM		
q_e_ (mg g^−1^)	1.34	1.62
k_2_ (mg min^−1^ g^−1^)	0.99	1.21
χ^2^	0.015	0.011
Adj. R^2^	0.7048	0.7637
BIC	−11.69	−13.99
	EKM	
RB		
α (mg g^−1^ min^−1^)	1.008	0.745
β (g mg^−1^)	0.248	0.557
χ^2^	2.58	0.14
Adj. R^2^	0.9041	0.9773
BIC	−19.39	−20.93
CHP		
α (mg g^−1^ min^−1^)	2.29	0.35
β (g mg^−1^)	3.94	1.66
χ^2^	0.117	0.080
Adj. R^2^	0.6083	0.9099
BIC	−23.78	−25.63
AZM		
α (mg g^−1^ min^−1^)	102.78	2063.37
β (g mg^−1^)	7.89	8.37
χ^2^	0.0045	0.006
Adj. R^2^	0.9143	0.8769
BIC	−31.21	−29.54
	IPD	
RB		
I part		
C (mg g^−1^)	−1.29	−0.0047
k_id_ (mg g^−1^ min^−0.5^)	2.028	0.855
R^2^	0.8760	0.9920
II part		
C (mg g^−1^)	8.42	6.53
k_id_ (mg g^−1^ min^−0.5^)	0.482	0.0042
R^2^	-	-
III part		
C (mg g^−1^)	12.14	-
k_id_ (mg g^−1^ min^−0.5^)	0.0012	-
R^2^	-	-
CHP		
I part		
C (mg g^−1^)	−0.816	−0.687
k_id_ (mg g^−1^ min^−0.5^)	0.988	0.665
R^2^	-	0.8746
II part		
C (mg g^−1^)	1.413	0.886
k_id_ (mg g^−1^ min^−0.5^)	−0.0063	0.179
R^2^	0.8268	0.7681
III part		
C (mg g^−1^)	-	2.293
k_id_ (mg g^−1^ min^−0.5^)	-	0.0091
R^2^	-	-
AZM		
I part		
C (mg g^−1^)	0.741	0.941
k_id_ (mg g^−1^ min^−0.5^)	0.118	0.178
R^2^	0.9975	0.8856
II part		
C (mg g^−1^)	1.372	1.398
k_id_ (mg g^−1^ min^−0.5^)	0.0016	0.0436
R^2^	−0.0736	-
III part		
C (mg g^−1^)	-	1.606
k_id_ (mg g^−1^ min^−0.5^)	-	0.0056
R^2^	-	-

**Table 2:** Isotherm parameters for contaminant adsorption using HCK and PHC at 22 °C.

Material	HCK			PHC		
C.	CHP	AZM	RB	CHP	AZM	RB
Freundlich isotherm model						
K_F_ ((dm^3^ mg^−1^)^1/n^)	0.184 ± 0.003	0.317 ± 0.004	2.06 ± 0.07	0.205 ± 0.001	0.319 ± 0.001	1.59 ± 0.02
n	1.63 ± 0.03	3.17 ± 0.04	1.26 ± 0.06	1.12 ± 0.01	1.71 ± 0.01	1.88 ± 0.02
χ^2^	0.015	0.004	33.222	0.003	0.004	0.816
Adj. R^2^	0.965	0.956	0.931	0.999	0.995	0.980
BIC	13.16	5.22	20.44	1.54	5.05	−1.79
Langmuir isotherm model						
K_L_ (dm^3^ mg^−1^)	0.057 ± 0.001	0.41 ± 0.01	0.0112 ± 0.0005	0.0062 ± 0.0001	0.065 ± 0.001	0.0073 ± 0.0001
q_max_ (mg g^−1^)	2.34 ± 0.01	0.94 ± 0.02	132 ± 6	30.1 ± 0.2	3.40 ± 0.01	55.6 ± 0.3
χ^2^	0.003	0.010	23.150	0.008	0.008	1.192
Adj. R^2^	0.993	0.898	0.952	0.998	0.991	0.952
BIC	11.54	5.19	18.27	5.56	6.55	0.47
Temkin isotherm model						
K_T_ (dm^3^ mg^−1^)	1.9 ± 0.4	12.5 ± 0.6	0.215 ± 0.003	1.7 ± 0.6	3.6 ± 0.8	0.387 ± 0.006
b_T_ (J g mol^−1^ mg^−1^)	7600 ± 100	17,000 ± 1000	123 ± 5	2900 ± 900	6000 ± 1000	534 ± 7
χ^2^	0.067	0.009	15.357	0.873	0.171	1.932
Adj. R^2^	0.843	0.906	0.968	0.733	0.795	0.953
BIC	21.34	23.99	15.81	16.46	20.85	3.37
Dubinin–Radushkevich isotherm model						
q_DR_ (mg g^−1^)	1.42 ± 0.05	0.82 ± 0.09	55.9 ± 0.6	5.45 ± 0.05	2.0 ± 0.3	15 ± 3
K_DR_ (mol^2^ J^−2^)	(3.52 ± 0.06) × 10^−6^	(5.90 ± 0.40) × 10^−7^	(3.40 ± 0.04) × 10^−5^	(3.51 ± 0.06) × 10^−5^	(2.20 ± 0.30) × 10^−6^	(1.70 ± 0.60) × 10^−5^
E (J mol^−1^)	380 ± 10	920 ± 90	120 ± 20	120 ± 30	500 ± 100	200 ± 100
χ^2^	0.022	0.019	13.923	0.142	0.099	13.129
Adj. R^2^	0.950	0.807	0.971	0.957	0.882	0.681
BIC	18.04	18.13	15.23	18.96	19.30	14.87

**Table 3:** Isotherm parameters for contaminant adsorption using HCK and PHC at 30 °C.

Material	HCK			PHC		
Contaminant	CHP	AZM	RB	CHP	AZM	RB
Freundlich isotherm model						
K_F_ ((dm^3^ mg^−1^)^1/n^)	0.282 ± 0.006	0.117 ± 0.007	2.4 ± 0.2	0.427 ± 0.001	0.093 ± 0.002	5.25 ± 0.05
n	1.68 ± 0.05	1.62 ± 0.08	1.4 ± 0.1	1.43 ± 0.01	1.09 ± 0.02	2.19 ± 0.04
χ^2^	0.050	0.010	48.679	0.022	0.011	10.671
Adj. R^2^	0.943	0.942	0.886	0.994	0.986	0.943
BIC	−14.29	−10.19	−20.31	−18.43	−8.46	−27.81
Langmuir isotherm model						
K_L_ (dm^3^ mg^−1^)	0.0681 ± 0.0002	0.0665 ± 0.0002	0.0158 ± 0.0008	0.0354 ± 0.0003	0.0108 ± 0.0002	0.0453 ± 0.0004
q_max_ (mg g^−1^)	3.15 ± 0.02	1.39 ± 0.02	100 ± 1	8.91 ± 0.01	9.15 ± 0.02	48.8 ± 0.5
χ^2^	0.012	0.003	33.859	0.002	0.008	8.147
Adj. R^2^	0.986	0.985	0.921	0.999	0.991	0.957
BIC	−21.27	−16.59	−28.17	−30.78	−16.73	−23.48
Temkin isotherm model						
K_T_ (dm^3^ mg^−1^)	1.9 ± 0.3	1.7 ± 0.2	0.211 ± 0.001	2.1 ± 0.7	1.3 ± 0.6	0.483 ± 0.004
b_T_ (J g mol^−1^ mg^−1^)	5400 ± 500	12,000 ± 2000	130 ± 8	2700 ± 300	6000 ± 1000	244 ± 6
χ^2^	0.137	0.022	5.671	0.738	0.199	8.326
Adj. R^2^	0.845	0.870	0.987	0.802	0.752	0.956
BIC	−9.26	−6.28	−12.83	−0.85	−0.13	−0.01
Dubini–Radushkevich isotherm model						
q_DR_ (mg g^−1^)	2.02 ± 0.03	0.872 ± 0.004	50.4 ± 0.3	4.0 ± 0.3	2.42 ± 0.04	34 ± 7
K_DR_ (mol^2^ J^−2^)	(2.91 ± 0.04) × 10^−6^	(2.83 ± 0.05) × 10^−6^	(2.46 ± 0.02) × 10^−5^	(2.74 ± 0.09) × 10^−6^	(2.51 ± 0.03) × 10^−5^	(1.1 ± 0.4) × 10^−5^
E (J mol^−1^)	415 ± 8	420 ± 7	143 ± 5	430 ± 10	141 ± 2	200 ± 100
χ^2^	0.024	0.004	12.202	0.377	0.009	42.171
Adj. R^2^	0.973	0.976	0.971	0.899	0.989	0.775
BIC	−17.92	−17.32	−18.52	−4.22	−7.92	−9.11

**Table 4:** Isotherm parameters for contaminant adsorption using HCK and PHC at 35 °C.

Material	HCK			PHC		
Contaminant	CHP	AZM	RB	CHP	AZM	RB
Freundlich isotherm model						
K_F_ ((dm^3^ mg^−1^)^1/n^)	0.407 ± 0.005	0.117 ± 0.007	8.52 ± 0.05	1.75 ± 0.04	0.089 ± 0.001	4.62 ± 0.03
n	1.75 ± 0.07	1.64 ± 0.08	1.59 ± 0.04	0.706 ± 0.004	1.11 ± 0.05	1.51 ± 0.02
χ^2^	0.114	0.013	1846.16	0.161	0.012	582.86
R^2^	0.927	0.921	0.944	0.963	0.983	0.968
BIC	−22.45	−21.05	−26.48	−21.75	−21.51	−26.47
Langmuir isotherm model						
K_L_ (dm^3^ mg^−1^)	0.0792 ± 0.0002	0.0702 ± 0.0003	0.00321 ± 0.00005	0.0794 ± 0.0001	0.0116 ± 0.0003	0.00222 ± 0.00001
q_max_ (mg g^−1^)	3.98 ± 0.02	1.34 ± 0.04	750 ± 6	6.91 ± 0.01	7.46 ± 0.05	620 ± 4
χ^2^	0.032	0.004	1145.56	0.031	0.008	155.77
Adj. R^2^	0.980	0.973	0.965	0.993	0.989	0.991
BIC	−29.11	−26.43	−22.25	−23.79	−23.66	−23.59
Temkin isotherm model						
K_T_ (dm^3^ mg^−1^)	2.1 ± 0.4	1.7 ± 0.3	0.10 ± 0.03	3.1 ± 0.7	1.3 ± 0.6	0.10 ± 0.03
b_T_ (J g mol^−1^ mg^−1^)	4200 ± 300	13,000 ± 2000	26 ± 4	2800 ± 800	6000 ± 2000	40 ± 10
χ^2^	0.249	0.023	5476.88	0.852	0.167	4135.97
Adj. R^2^	0.840	0.860	0.834	0.803	0.756	0.774
BIC	−18.45	−18.20	−22.65	−7.39	−8.28	−8.15
Dubini–Radushkevich isotherm model						
q_DR_ (mg g^−1^)	2.67 ± 0.05	0.862 ± 0.005	420 ± 20	4.41 ± 0.05	2.20 ± 0.06	356 ± 8
K_DR_ (mol^2^ J^−2^)	(2.35 ± 0.05) × 10^−6^	(2.84 ± 0.04) × 10^−6^	(5.00 ± 0.2) × 10^−4^	(1.63 ± 0.05) × 10^−6^	(2.33 ± 0.03) × 10^−5^	(2.87 ± 0.06) × 10^−3^
E (J mol^−1^)	461 ± 9	419 ± 7	32 ± 4	553 ± 9	146 ± 6	13.2 ± 0.7
χ^2^	0.027	0.003	3679.04	0.180	0.007	1341.52
Adj. R^2^	0.982	0.983	0.888	0.959	0.989	0.927
BIC	−26.94	−28.87	−19.07	−22.93	−23.92	−10.91

**Table 5:** Comparison of different biomass and carbon materials as adsorbents of CHP, AZM, and RB.

Pollutants	Materials	q_max_ (mg g^−1^)	pH	Contact Time (min)	Ref.
RB	*Rhus coriaria* L. plant	37.93	3.0	180	[13]
	Coconut coir	13.00	7.0	60	[51]
	*Calophyllum inophyllum* seeds biochar	169.5	2.0	25	[7]
	MoS_2_ nanosheet fungus residue biochar	102.00	-	120	[28]
	*Atropa belladonna*@ZnCl_2_	263.19	6.0	120	[20]
	Citric acid-modified furfural residue hydrochar	39.46	3.0	120	[21]
	*Parthenium hysterophorus* biochar	14.90	6.0	60	[53]
	HCK PHC	751.0 616.0	6.0	120	This study
CHP	Rice husk biochar Date pit biochar Sugarcane bagasse biochar	0.082 0.323 0.304	5.0 3.0 3.0	60 120 120	[50]
	Sunflower	1.97	-	120	[52]
	Irradiated plum pomace biochar	0.428	-	60	[54]
	KOH-spent coffee grounds biochar	16.10	6.0	1440	[55]
	Viscose textile biochar	12.8	6.0	120	[6]
	HCK PHC	3.98 30.10	6.0	120	This study
AZM	Horseshoe crab biochar	8.26	6.5	500	[56]
	Peat moss biochar	4.54	6.5	-	[10]
	Viscose textile biochar	6.56	6.0	120	[6]
	HCK PHC	1.39 9.15	6.0	120	This study

**Table 6:** Thermodynamic parameters of CHP, AZM, and RB adsorption onto materials at 22, 30, and 35 °C.

		ΔH^0^ (kJ mol^−1^)	ΔS^0^ (J mol^−1^K^−1^)	ΔG^0^ (kJ mol^−1^)			R^2^
	T (°C)			22	30	35	
CHP	HCK	36.8 ± 0.5	156 ± 5	−9.30 ± 0.08	−10.5 ± 0.2	−11.3 ± 0.2	0.985
	PHC	6.39 ± 0.04	62.8 ± 0.7	−12.1 ± 0.9	−12.7 ± 0.9	−13.0 ± 0.9	0.953
AZM	HCK	−4.97 ± 0.01	11.7 ± 0.1	−8.41 ± 0.01	−8.51 ± 0.01	−8.57 ± 0.01	0.993
	PHC	−12.0 ± 0.2	−4.41 ± 0.06	−10.7 ± 0.3	−10.6 ± 0.3	−10.6 ± 0.3	0.990
RB	HCK	42.9 ± 0.5	200 ± 8	−16.2 ± 0.3	−17.8 ± 0.4	−18.8 ± 0.4	0.00913
	PHC	102 ± 8	389 ± 6	−12.7 ± 0.5	−15.8 ± 0.6	−17.7 ± 0.7	0.98017

## Data Availability

The data presented in this study are available upon request from the corresponding author.

## Supplementary Materials

The following supporting information can be downloaded at: https://www.mdpi.com/article/10.3390/ijms27072984/s1.

## Abbreviations

The following abbreviations are used in this manuscript:

AZM	Azinphos-methyl pesticide
BET	Brunauer–Emmett–Teller analysis
CHP	Chlorpyrifos pesticide
EKM	Elovich kinetic model
FTIR	Fourier-Transform Infrared spectroscopy
GP	Grape pomace
HCK	Potassium hydroxide-activated grape pomace hydrochar
HTC	Hydrothermal carbonization
IPD	Intraparticle diffusion model
PFO	Pseudo-second-order kinetic model
PHC	Pyrolized grape pomace hydrochar
pHpzc	Point of zero charge
PSD	Particle size distribution
PSO	Pseudo-first-order kinetic model
RB	Rhodamine B dye
SEM-EDX	Scanning Electron Microscopy coupled with Energy-Dispersive X-ray Spectroscopy
