# Supplementary material for: Sustainable Hydrochars from Winery Waste for the Efficient Removal of Organophosphorus Pesticides and Synthetic Dye
Source: Int J Mol Sci. 2026 Mar 25;27(7):2984. doi: 10.3390/ijms27072984 (PMC13073505; doi:10.3390/ijms27072984)
Supplement: Supplementary file 1 [file ijms-27-02984-s001.zip › ijms-4175279-supplementary.pdf]

Supplementary Materials

Table S1. Summary of Applied Adsorption Models, Their Equations, and Fitted Parameters.

	Models	Equations	Parameters
**Adsorption isothermal model**	Langmuir	$q_t = \frac{q_{max} K_L C_e}{1 + K_L C_e}$	q_t (mg g^{-1}) quantity of adsorbed pollutant for a specified Ce<br>q_{max} (mg g^{-1}) maximum adsorption capacity<br>K_L (dm^3 mg^{-1}) Langmuir isotherm constant<br>C_e (mg dm^{-3}) equilibrium concentration
	Freundlich	$q_t = K_F C_e{}^{1/n}$	K_F ((mg g^{-1})(dm^3 g^{-1})$^{1/n}$) Freundlich isotherm constant<br>1/n factor of heterogeneity
	Temkin	$q_t = \frac{RT}{b_T} lnK_T C_e$	K_T (dm^3 mg^{-1}) Temkin isotherm equilibrium binding constant<br>T (K) temperature<br>R (J $mol^{-1}K^{-1}$) universal gas constante<br>b_T (J g mol^{-1} mg^{-1}) Temkin isotherm constant
	Dubinin–Radushkevich	$q_t = q_{DR} e^{-K_{DR}\varepsilon^2}$	q_{DR} (mg g^{-1}) theoretical isotherm saturation capacity<br>K_{DR} (mol^2 J^{-2}) Redlich-Peterson isotherm constant<br>ε (J mol^{-1}) Polanyi potential
**Kinetic isothermal model**	PFO	$q_t = q_e(1 - e^{-k_1 t})$	q_t(mg g^{-1}) quantity of removal pollutant at t(min)<br>q_e(mg g^{-1}) equilibrium adsorption amount<br>k_1 (min^{-1}) PFO adsorption rate constant
	PSO	$q_t = \frac{k_2 q_e^2 t}{1 + k_2 q_e t}$	k_2 (mg $g^{-1}min^{-1}$) PSO adsorption rate constant
	EKM	$q_t = \frac{1}{\beta}(1 + \alpha\beta t)$	α (mg g^{-1} min^{-1}) initial adsorption rate<br>β(g mg^{-1}) desorption constant
	IPD	$q_t = k_{id} t^{0.5} + C$	C (mg g^{-1}) boundary layer constant

			k_{id} (mg·g^{-1}·min$^{1/2}$) intraparticle diffusion rate constant
**Thermodynamics**	Gibbs-Helmholtz	$\Delta G^0 = \Delta H^0 - T\Delta S^0$	ΔG^o (kJ·mol^{-1}) standard free energy change<br>ΔH° (kJ·mol^{-1}) standard enthalpy change<br>ΔS° (J·mol^{-1}·K^{-1}) entropy change
	Van't Hoff equation	$lnK^0_{dist} = -\frac{\Delta H^0}{RT} + \frac{\Delta S^0}{R}$<br>$K^0_{dist} = \frac{q_e}{C_e} \cdot \frac{C^0}{q^0}$<br>$\Delta G^0 = -RTlnK^0_{dist}$	

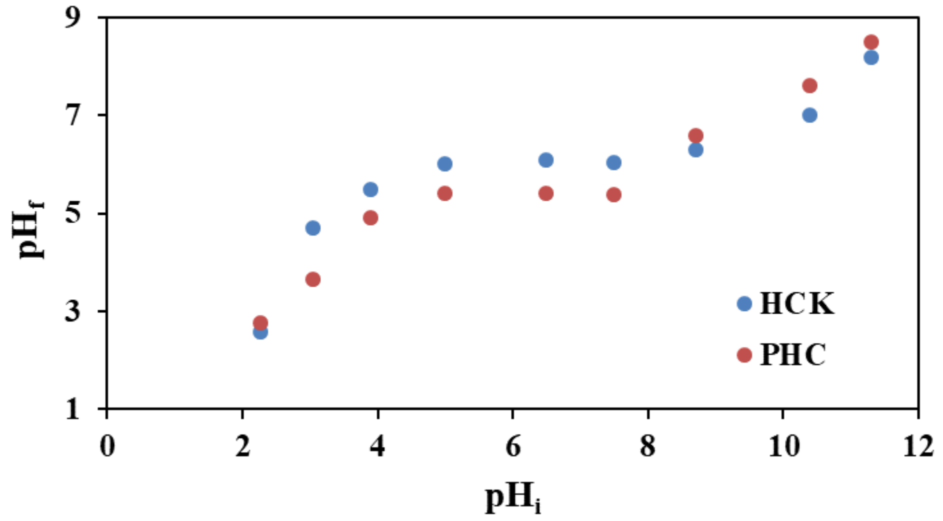

Figure S1. pH_i vs. pH_f curves for HCK and PHC, indicating their pH_{PZC}

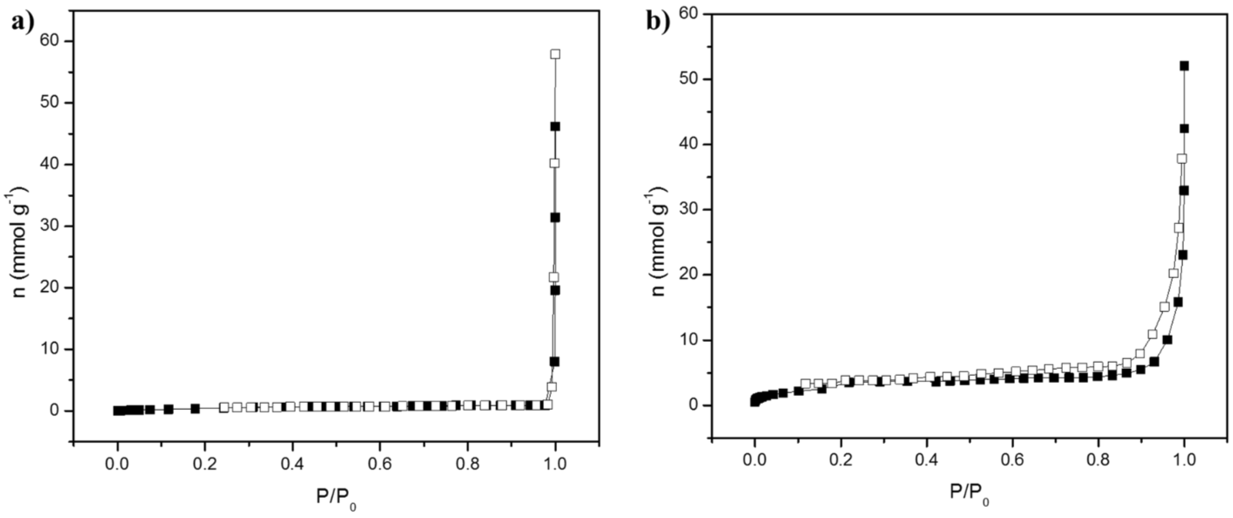

Figure S2. Nitrogen adsorption isotherm plot for samples HCK (a) and PHC (b). Solid symbols – adsorption, open symbols – desorption.

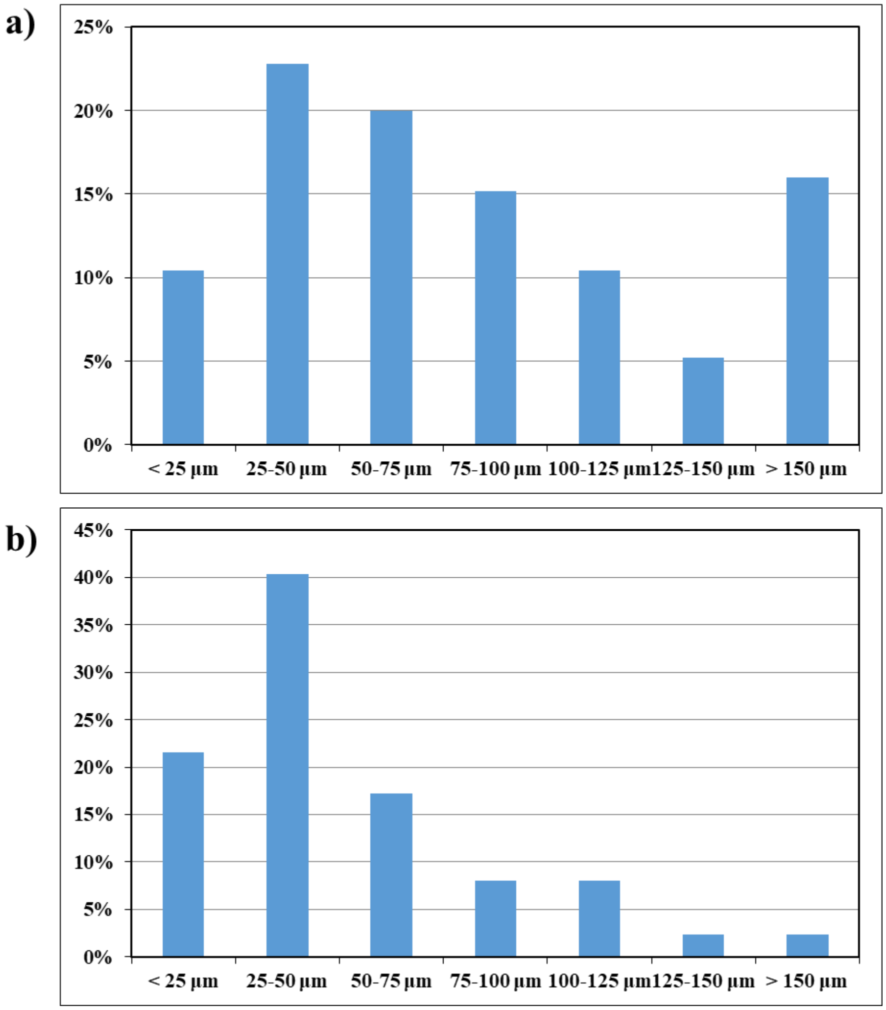

Figure S3. Particle size distribution of HCK (a) and PHC (b) Hydrochars

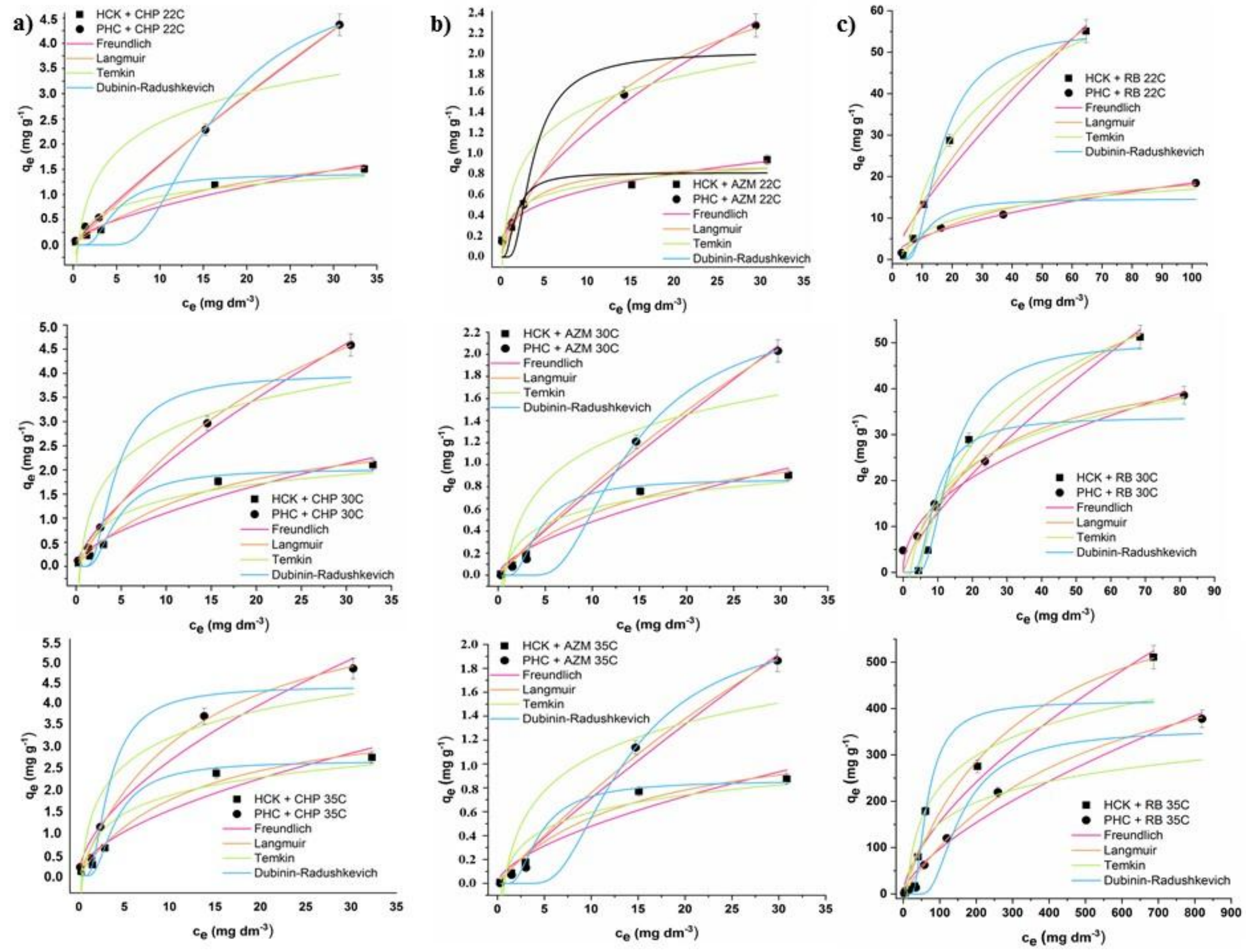

Figure S4.Non-linear isotherm model fits (mean ± SD, n = 3) for adsorption of (a) CHP, (b) AZM, and (c) RB onto HCK and PHC at 22, 30, and 35 °C (1 mg mL^{-1}, 120 min, pH=6).

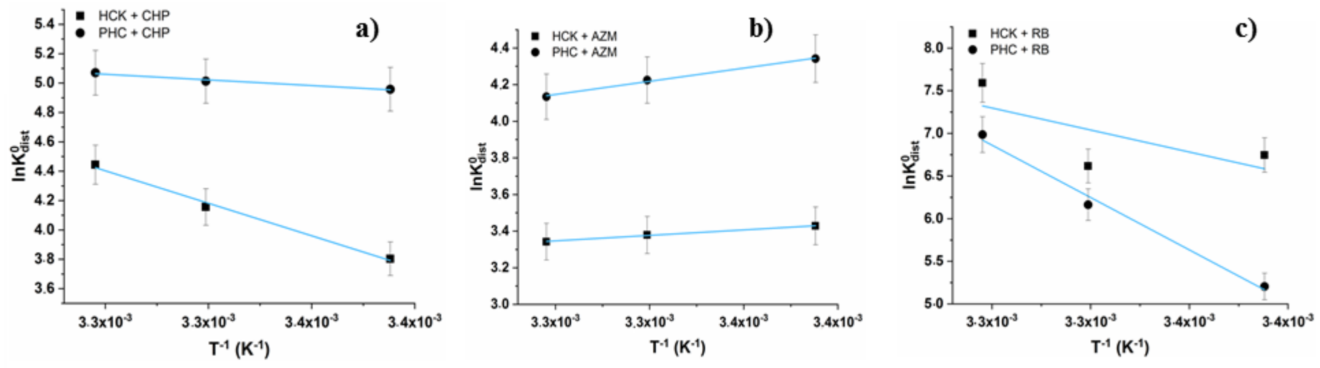

Figure S5. Van't Hoff plots for (a) HCK and PHC after CHP, (b) HCK and PHC after AZM, and (c) HCK and PHC after RB adsorption at 22, 30, and 35 °C.

Table S2. Estimated cost analysis of synthesized hydrochars (USD)

Cost description	HCK	PHC	Remarks
Electricity (HTC)	0.05 USD	0.05 USD	1 h at 220 °C (400 W autoclave)
Electricity (pyrolysis)	-	0.12 USD	1 h at 400 °C (1 kW furnace)
KOH	0.32 USD	-	Analytical grade KOH
Filter papers	0.29 USD	0.10 USD	3 pcs for HCK; 1 pc for PHC
Water	negligible	negligible	Distilled water produced in lab
Biomass	0	0	Waste biomass, freely available
Total cost (1 kg)	≈ 32.8 USD kg^{-1}	≈ 13.3 USD kg^{-1}	Corresponding to 1 kg of product
